# Effect of Dietary Interventions on Body Composition and Quality of Life in Stomach Cancer Survivors after Gastrectomy: A Systematic Review

**DOI:** 10.1007/s12029-025-01388-5

**Published:** 2026-01-13

**Authors:** Abhishek Anand, T. R. Dilip, Leone Craig, Manish Bhandare, Sara MacLennan, Aravinda Meera Guntupalli

**Affiliations:** 1 International Institute for Population Sciences, Mumbai, India; 2 Department of Surgical Oncology, Tata Memorial Hospital, Mumbai, India; 3 School of Medicine, Medical Sciences and Nutrition (SMMSN), University of Aberdeen, Aberdeen, Scotland

**Keywords:** Gastric cancer, Gastrectomy, Nutritional interventions, Comprehensive nutritional counselling, Body composition, Quality of life

## Abstract

**Purpose:**

Nutritional interventions are critical in supporting recovery and maintaining nutritional status in gastric cancer patients post-gastrectomy. This systematic review evaluates the impact of various dietary interventions on outcomes such as body composition, body weight loss (BWL) and quality of life (QoL) of patients with gastrectomy.

**Methods:**

A comprehensive search was conducted across six electronic databases, restricted to studies published in English after January 2000 that involved human subjects. Data were synthesised descriptively due to the heterogeneity of study designs and interventions. Of the 2103 studies identified, 13 met the inclusion criteria. These studies included a range of interventions such as Oral Nutritional Supplements (ONS), nutritional counselling, and dietary education.

**Results:**

The results demonstrated that ONS and nutritional counselling significantly reduced BWL, particularly in total gastrectomy patients. Evidence for improvements in QoL was more limited, with only one study reporting a statistically significant effect, and considerable heterogeneity in QoL tools and follow-up durations. Simplified dietary education, without follow-up, showed limited effectiveness. Nutritional counselling and ONS significantly improve postoperative outcomes in gastric cancer patients. Personalised, continuous interventions reduce BWL, preserve skeletal muscle, and enhance QoL.

**Conclusion:**

Integrating dietitians into care, especially in low-resource settings, is vital. Standardised assessments and stratified research by gastrectomy type are essential for effective, tailored interventions.

**Supplementary Information:**

The online version contains supplementary material available at 10.1007/s12029-025-01388-5.

## Introduction

Gastric cancer remains a significant health challenge globally due to its rising prevalence and mortality rates [1]. It ranks as the fifth most prevalent cancer and the third leading cause of cancer-related mortality worldwide [2]. It accounts for approximately 6% of all cancer diagnoses and around 770,000 deaths recorded globally in 2020 [3, 4]. Curative treatment of gastric cancer involves the surgical intervention of removing the stomach, either fully or partially, known as gastrectomy. Despite the invasiveness of this procedure, it remains a critical component of curative treatment, often complemented by adjunctive therapies such as chemotherapy, radiotherapy and immunotherapy to terminate cancer cells and reduce cancer recurrence risks [5].

Removing part of the stomach or the full stomach will likely result in a range of complications, such as reduced food intake and nutritional malabsorption [6]. Postoperative individuals frequently experience challenges such as early satiety, altered digestion, and malabsorption of essential nutrients, which leads to weight loss and deficiencies in vitamins and minerals [7]. Additionally, patients may experience dumping syndrome, with symptoms such as diarrhoea, nausea and exhaustion due to rapid gastric emptying, which further complicates post-surgical nutritional behaviours [8]. Poor dietary intake and accompanying malnutrition following a gastrectomy are closely associated with a higher risk of postoperative complications, such as body weight loss (BWL) and weakening of the immune system, which makes patients more susceptible to infections. These adverse outcomes often result in prolonged hospital stays, further delaying recovery and reducing Quality of Life (QoL). All these increase the burden on individuals and the healthcare system. Existing research suggests that nutritional interventions could play an essential role in mitigating these impacts for cancer patients. The evidence regarding the effectiveness of nutritional intervention in supporting gastric cancer patients, however, is not conclusive.

A review evaluating the effects of the duration of hospital stay and disease-related complications among gastric cancer patients following dietary interventions suggests a positive impact [9]. The Taiwan consensus on nutritional support project further emphasises that early oral feeding can result in a shorter hospital stay for gastrectomy patients. This study also recommends tailored nutrition management for patients undergoing gastrectomy to address nutritional challenges [10]. A few more studies have also assessed the impact of nutritional and dietary interventions on other health outcomes among individuals with gastrectomy. For example, in a South Korean study, dietary interventions enhanced nutritional and functional status post-gastrectomy [11]. Additionally, post-discharge nutritional support, including oral supplements and dietary advice, was shown to be beneficial in maintaining post-surgery nutritional status [12].

All these studies individually highlight the importance of nutritional or dietary support measures to reduce complications post-gastrectomy. However, there is a need for a comprehensive, evidence-based recommendation on the effect of dietary interventions on body composition and QoL post-gastrectomy. This requires review and pooling outcomes from existing studies of nutritional interventions for gastrectomy patients from multi-cultural settings with diverse dietary behaviours. This exercise is part of the World Cancer Research Fund-supported EASE-IN collaboration working on the feasibility and acceptability of conducting a randomised controlled trial to improve the body composition and QoL of stomach cancer patients after total gastrectomy. As a first step, a systematic review of all globally available nutritional interventions for gastrectomy patients was conducted to consolidate the existing evidence on interventions and treatment outcomes. The results of this systematic review are expected to provide inputs for drafting nutritional guidelines for patients subjected to gastrectomy and inform future interventions and practices on body composition and QoL in stomach cancer survivors after gastrectomy to support these patients.

## Materials and Methods

### Search Strategy and Selection Criteria

In this systematic review, a comprehensive search was conducted across the following electronic databases: MEDLINE, EMBASE, Web of Science, ProQuest, CINAHL, and the Cochrane Library, with the following restrictions: English language, publications from January 2000 onwards, and studies involving human subjects only. The initial search on the database was conducted for publications up to July 9, 2023; the search was last updated on September 11, 2024. The study conforms to the Preferred Reporting Items for Systematic Reviews and Meta-Analysis (PRISMA) guidelines [13]. Ethical approval was not required as the study participants were drawn from previously published research.

The search terms included terms and synonyms related to gastric or stomach cancer, gastrectomy, and dietary interventions. The methodology included clinical trials, pilot and feasibility studies. The essential synonyms and phrases were used both separately and in conjunction with Boolean operators “AND” and “OR” (Supplementary Table 1). The articles were uploaded to the Covidence systematic review platform to handle citations and expedite the review process [14]. Studies were considered eligible if they met the inclusion criteria listed in Table 1.

**Table 1:** Inclusion and exclusion criteria

	Inclusion Criteria	Exclusion criteria
Population	Adult cancer patients aged > 18 years who underwent partial or total gastrectomy and chemotherapy due to stomach cancer	• Very frail (Challenges performing basic activities of daily living) • Gastric cancer, along with secondary cancers in the past two years • Patients with severe frailty or dementia • Patients with radiotherapy post gastrectomy • Patients with removal of other organs along with stomach
Intervention	• Dietary (food & nutrional counselling)	• Trials on pharmacological interventions and vitamin supplementation • Nutrition intervention in combination with pharmacological intervention
Outcome	• Any body composition indicator, including body mass index, body weight, and body fat (Primary) • Quality of life (Secondary)	
Study design	• Randomised control trials (RCT) • Case control	• Observational studies, cohort studies, and cross-sectional studies
Publication type	• Published afer the year 2000 • Studies published in the English language • Full free text availability	• Conference abstracts • Reviews of all types • Grey literature

After removing duplicate articles, the studies were independently screened on Covidence, primarily by two authors (AA, AG), with a small share screened by a project team member (SC). The first selection included screening based on title and abstracts, followed by full-text screening. Any conflicts between these authors were resolved with the help of a fourth team member (DTR). Various nutritional intervention types were included, including dietary interventions, Oral Nutritional Supplements (ONS) and nutritional counselling. Due to the heterogeneity of studies, this systematic review is limited to descriptive synthesis only.

## Study Selection and Characteristics

**Fig. 1:**
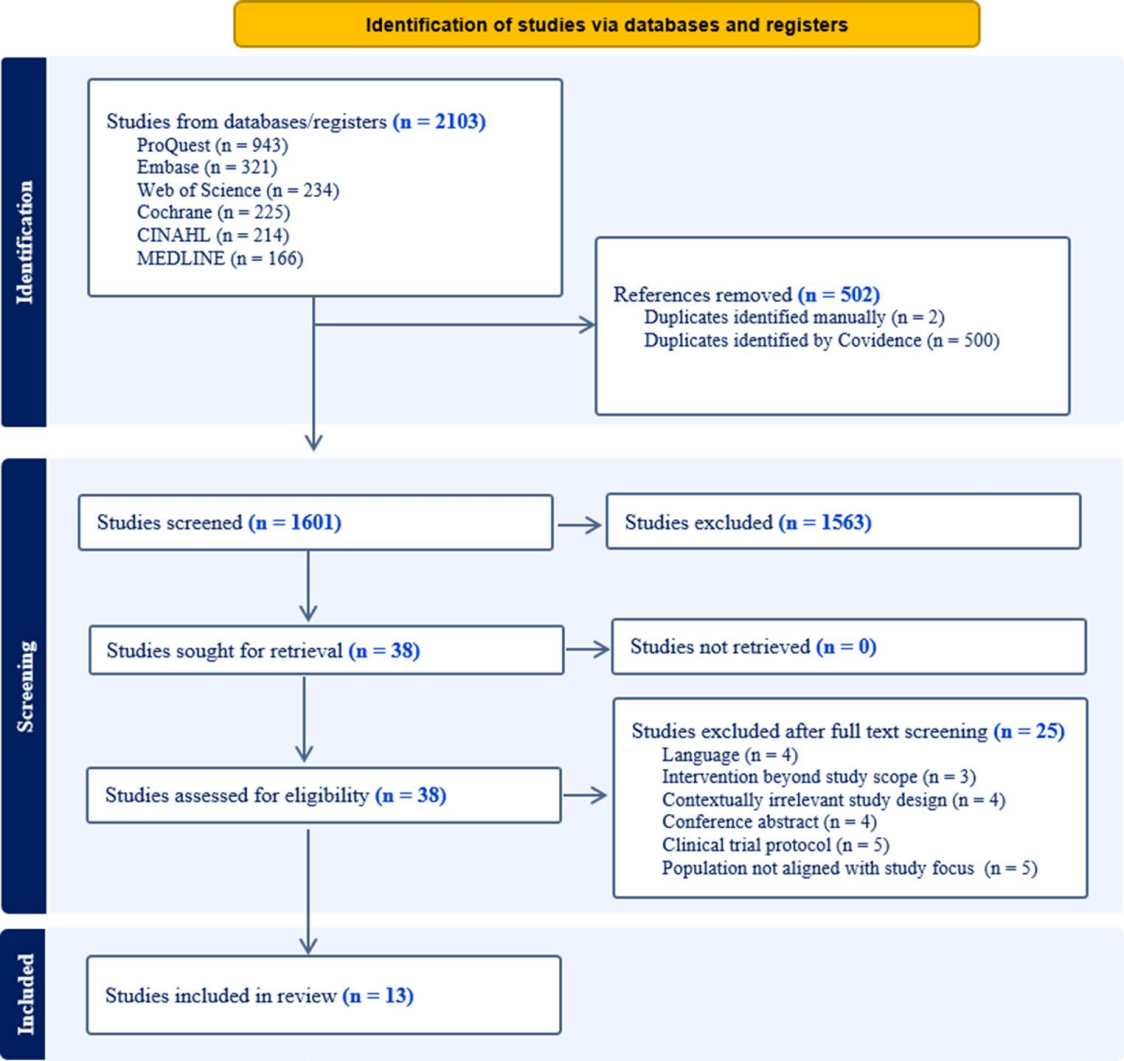
Prisma flowchart for the study selection process

The literature search yielded 2103 studies from six electronic databases; duplicate studies (*n* = 502) and irrelevant studies (*n* = 1563) were excluded based on title and abstract screening. Finally, 13 studies that met our inclusion criteria were included in the review (Fig. 1).

## Quality Assessment

**Fig. 2:**
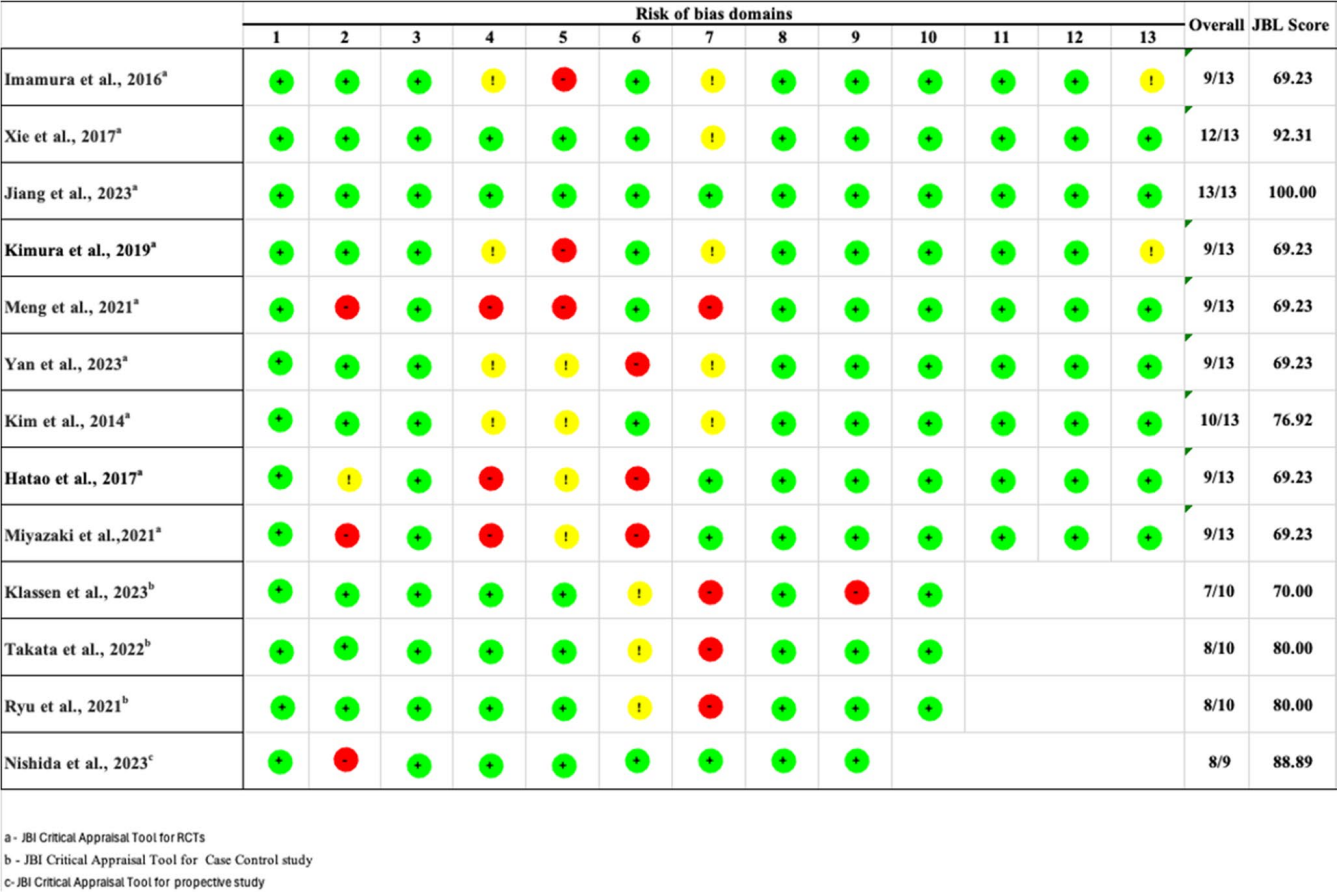
Risk of bias assessment

We used the Joanna Briggs Institute (JBI) critical appraisal tool to evaluate the quality of RCTs, case-control and prospective studies [15–17]. Two authors (AA and AG) independently assessed the quality of the studies and calculated the scores based on the JBI checklist, and a third author (DTR) later verified the assessment (Fig. 2). The risk of bias was considered high when only 40–60% of the checklist questions received a “Yes” response. It was deemed moderate when 60–80% of the questions were answered “Yes”, and if 80–100% of the checklist questions were marked with “Yes,” the risk of bias was considered low. Across the 13 studies, 5 (38%) were of low risk of bias (≥ 80% JBI items met), 8 (62%) were of moderate risk (≥ 60% to < 80%), and none were of high risk (< 60%). The most frequent ‘No/Unclear’ ratings were in domains related to allocation and blinding, as well as confounder control, especially among non-randomised designs.

## Protocol Registration

The review was registered in the OSF registry: 10.17605/OSF.IO/K8TVF.

## Patient and Public Involvement

No patients or the public were involved in the preparation of this systematic review.

## Results

### Study Characteristics

Table 2 summarises the characteristics of all 13 studies on dietary interventions for stomach cancer survivors after gastrectomy. The intervention studies are predominantly randomised trials (*n* = 9), followed by case-control studies (*n* = 3) and prospective studies (*n* = 1), respectively. Sample sizes varied, with most studies involving 100–199 participants (*n* = 5). Geographically, the studies are concentrated in East Asia, particularly China (*n* = 4), Japan (*n* = 5), and South Korea (*n* = 2). The interventions comprised various approaches, including ONS alone (*n* = 4), nutritional counselling alone (*n* = 2), a combination of nutritional counselling and ONS (*n* = 2), nutritional counselling with education (*n* = 3), an integrated approach involving ONS, nutritional counselling and physical activity (*n* = 1), and education-based intervention alone (*n* = 1). The interventions typically lasted more than 10 weeks, with a maximum duration of up to 48 weeks. Most studies include patients with total and distal gastrectomy, with only one study explicitly addressing distal gastrectomy; for more details, refer to Table 3.

**Table 2:** Characteristics of studies reporting dietary interventions in stomach cancer survivors after gastrectomy

Characteristics	*N* = 13
Study type	
Randomised clinical trial	9
Case control	3
Prospective study	1
**Sample size**	
11–49	3
50–99	2
100–199	5
200+	3
**Country and region**	
China	4
Japan^*^	6
Germany	1
South Korea	2
Taiwan^*^	1
**Mode of intervention**	
Mobile app based	1
Face-to-face	9
Mixed^#^	3
**Type of intervention**	
ONS	4
Nutritional counselling (NC)	2
Education	1
NC + Education	3
NC + ONS	2
ONS + NC + Physical activity	1
**Duration of intervention**	
≤ 5 weeks	3
6–8 weeks	2
12 weeks	6
48 weeks	2
**Type of gastrectomy**	
Distal only	1
Distal and Total	6
Partial and Total	2
Distal, Proximal and Total	2
Unspecified	2

*- One study included participants from Japan and Taiwan,* # - Mixed includes face to face and tele-counselling both*

**Table 3:** Characteristics of studies reporting nutrition interventions

Authors, Year	Country	Sample Size	Main Inclusion Criteria	Duration of intervention	Intervention	Nutritional Status indicators	Quality of Life (QoL)
Kim et al., [11]	South Korea	48	• Diagnosed with stomach cancer • Underwent total or partial gastrectomy • Aged over 20 years • No evidence of metastasis • No severe physical or mental illnesses	6 weeks	• Education • Nutritional counselling • Patient participation based dietary intervention (PPDI)	• BWL was lower in the intervention group compared to the control group. • The intervention group showed significant reductions (*p* < 0.05) in adverse dietary symptoms over time. • There were notable improvements (*p* < 0.05) in: Functional status, performance status, dietary intake, adherence to dietary guidelines, dietary knowledge, satisfaction with the intervention	The QoL score among experimental group improved significantly over time. (T0–61.92 to T2–86.27) (*p* < 0.01)
Imamura et al., [18]	Japan	111	• Previously untreated (except for gastrectomy) histopathologically confirmed gastric adenocarcinoma • Aged over 20 years • Clinical stage I, II, or III disease • Eastern Cooperative Oncology Group performance status (PS) of 0–2 • Underwent curative resection • Able to take oral nutrition • No severe postoperative complications between surgery and randomisation.	6–8 weeks	• Oral Elemental Diet 300 mL/day (300 kcal/day)	• The BWL was significantly different between the ED (elemental diet) and control groups. • ED supplementation was effective in reducing postoperative weight loss in gastric cancer patients undergoing gastrectomy.	NR
Xie et al., [26]	China	144	• Pathologically confirmed diagnosis of gastric cancer • Underwent partial or total gastrectomy • Aged 18 years or older	< 4 weeks	• Educational intervention, • Nutritional prescription • Guideline for dealing with chemotherapy-induced side effects	The interventions significantly improved: • Calorie and iron intake within 24 h after the first chemotherapy session. • Patients’ weight, hemoglobin, total serum protein, and albumin levels throughout the chemotherapy course. • Chemotherapy compliance rate was significantly higher in the intervention group compared to the control group (73.61% vs. 55.56%, *p* = 0.024).	NR
Hatao et al., [20]	Japan, Taiwan	113	• Aged 20 years or older • Capable of consuming food orally • Scheduled for curative total or distal gastrectomy for gastric cancer treatment • No prior treatments for gastric cancer • No perioperative nutritional issues	12 weeks	• ONS	• Patients in the ONS group retained 88.5% of their preoperative body weight, compared to 85.6% in the control group (*p* < 0.05). • ONS reduced weight loss in patients who underwent total gastrectomy. • For patients with distal gastrectomy, no significant difference in weight loss was observed between the ONS and control groups.	No significant improvements between the intervention and control groups both groups experienced similar levels of QoL
Kimura et al., [19]	Japan	106	• Previously untreated (except for gastrectomy) • Histopathologically confirmed gastric adenocarcinoma • Aged 20 years or older • Clinical stage I, II, or III • Eastern Cooperative Oncology Group performance status of 0–2 • Underwent curative resection • Able to take oral nutrition • No severe postoperative complications between surgery and randomisation	6–8 weeks	• Elemental Diet (ED), ensure Liquid (ONS)	• One year after surgery, the BWL was significantly lower in the ED group compared to the control group among patients who underwent total gastrectomy. • For patients who underwent distal gastrectomy, there was no significant difference in BWL between the ED and control groups. • ED was the only factor found to impact BWL at one year post-surgery for TG patients. • A daily nutritional intervention of 300 kcal/day ED for 6–8 weeks effectively reduced BWL at both 6–8 weeks and one year post-surgery in TG patients.	NR
Meng et al., [12]	China	337	• Discharged after surgery for gastric cancer • Nutritional risk score of 3 or greater based on the Nutritional Risk Screening 2002 (NRS 2002) tool	12 weeks	• Dietary advice and an oral intake of 118 Nutren^®^ Optimum (Nestle Health Science, Switzerland) at a 500 mL daily dosage	Patients who received ONS along with dietary advice experienced: • Significantly less weight loss, • Higher BMI, higher skeletal mass index (SMI), compared to those who received dietary advice alone (*p* < 0.05). • The incidence of sarcopenia was also significantly lower in the ONS group (*p* < 0.05).	Regarding QoL, patients who received ONS and dietary advice reported significantly less fatigue and appetite loss than those who received dietary advice alone (*p* < 0.05). However, there were no significant differences between the two groups in other outcomes (*p* > 0.05).
Ryu et al., [24]	South Korea	200	• Underwent subtotal gastrectomy • Diagnosed with pathologically confirmed Stage I (T1N0, T1N1, and T2N0) gastric cancer according to the American Joint Cancer Classification (AJCC; 8th edition) • Did not receive postoperative adjuvant chemotherapy that might influence nutritional status • Had two years of body weight records and laboratory findings available	48 weeks	• Simplified dietary education	• No significant differences were observed between the two groups in clinical characteristics, and serological parameters. • Similarly, nutritional parameters, such as: • BWL, BMI change, and PNI, were not significantly different between the groups.	NR
Miyazaki et al., [21]	Japan	880	• Aged 20–85 years • Underwent DG, PG, or TG • No clinical metastasis • Adequate organ function • No postoperative complications	12 weeks	• ONS	At 3 months post-surgery: • BWL was significantly lower in the intervention group (IG) compared to the control group (CG): 7.1% vs. 8.5% (*p* = 0.0011). At 6 months post-surgery: • The IG continued to show significantly less BWL than the CG: 8.6% vs. 9.7% (*p* = 0.0228). At 1 year post-surgery: • The difference in BWL between the IG and CG diminished and became statistically non-significant: 9.3% vs. 9.8% (*p* = 0.37).	NR
Klassen et al., [23]	Germany	62	NA	4–6 weeks	• Nutritional counselling • If nutritional problems persists, high-caloric fluid supplements (2–3 potions (200 mL/day) and enteral or parenteral nutrition.	• No significant differences were observed between the groups in terms of BMI, NRS, complications, length of hospital stay, and mortality. • Within the intervention group (IG), there was a tendency to lose 1.74 kg of weight (*p* = 0.046), a decrease in phase angle by 0.59° (*p* = 0.004), and an increase in NRS by 1.34 points (*p* < 0.001) when comparing post- and preoperative parameters. • No significant effect of perioperative nutritional therapy in gastrointestinal cancer patients was found	NR
Nishida et al., [28]	Japan	18	• Aged 65 years or older • Underwent distal or total gastrectomy for gastric cancer with curative intent • Eastern Cooperative Oncology Group performance status of 0–1	4 weeks	• Oral nutritional supplements, • Individual nutritional counselling, • Protein (BCAA) rich supplements • Rehabilitation with physical therapists • Walking for more than 5000 steps/day • Resistance training included calf raises and squats.	• Postoperative exercise and nutritional therapies with BCAA-rich supplements may benefit elderly patients after gastrectomy by reducing the loss of SMI and mitigating decreases in QoL.	QoL scores showed almost the same degree of recovery at 1 month after gastrectomy as preoperative scores. (0.8855 before surgery, 0.7669 1 week after surgery, and 0.8338 1 month after surgery)
Jiang et al., [27]	China	24	• Histologically confirmed gastric adenocarcinoma • Received D2 radical gastrectomy • Access to broadband internet • Aged 18 years or older	12 weeks	• Nutrition counselling by 12-week individualised mHealth nutrition intervention (iNutrition intervention) using ordinary food and ONS advice	• The iNutrition intervention significantly enhanced participants’ nutritional behavior (*p* = 0.005), increased their energy intake (*p* = 0.038), and improved their adherence to both energy (*p* = 0.006) and protein (*p* = 0.008) requirements.	The QoL score was higher in the intervention group at T0, T1 and T2.
Takata et al., [22]	Japan	93	NA	48 weeks	• Perioperative and post-discharge nutritional counselling, • Low-carbohydrate, protein-rich, high-fat diet, and ONS was recommended for patients with insufficient energy intake	• The nutritional counselling group experienced significantly less BWL than the control group at 1 month, 6 months, and 12 months post-surgery. • Additionally, the nutritional counselling group had significantly less SML at 12 months. Changes in total cholesterol were also significantly lower in the nutritional counselling group compared to the control group at 12 months post-surgery (*p* = 0.02). • Not receiving nutritional counselling was an independent risk factor for severe BWL (*p* = 0.001) and %SML (*p* = 0.006) at 12 months post-surgery.	NR
Yan et al., [25]	China	130	• Aged 18–75 years • Discharged after radical gastrectomy • Underwent reconstruction of gastrointestinal tract function recovery, allowing food intake • Able to communicate without barriers.	8 weeks	• Personalised dietary counselling to achieve protein and calorie targets, • ONS	• Patients in the intervention group had significantly less BMI loss compared to the control group at 30 days, 60 days, and 90 days post-surgery (all *p* < 0.05). • Subgroup analysis by surgery type revealed that the intervention notably reduced BMI loss in patients undergoing total and proximal gastrectomy at 30 days, 60 days, and 90 days after surgery (all *p* < 0.05).	Regarding the QOL, at 90 days post-surgery, patients in the intervention group experienced significantly less fatigue, shortness of breath, and stomach pain, as well as improved physical and cognitive function (*p* < 0.05).

*NA: Not available*,* NR: not relevant*,* RCT – Randomised controlled trial*,* ONS – Oral Nutritional Supplements*

### Type of Interventions

The most common interventions were ONS, nutritional counselling and the ONS with nutritional counselling.

### Oral Nutritional Supplements

In this review, four studies used ONS such as oral elemental diets, high-caloric fluid supplements, and protein (Branched-chain amino acid (BCAA)) - rich supplements as interventions (Table 3). Imamura et al. [18] evaluated the effect of a low-fat (300 kcal/day) Elemental Diet (ED) with essential amino acids on BWL. They found that their 6–8 week intervention significantly reduced BWL in the group receiving ED at 6–8 weeks compared to the control group [18]. In their follow-up study by Kimura et al. [19], this intervention effectively reduced BWL even at one year post-gastrectomy [19]. Hatao et al. [20] included both distal (partial) and total gastrectomy patients in the intervention (ONS) and control groups. They reported that ONS (Concentrated liquid diet ANOM, 400 kcal/day) significantly reduced BWL in total gastrectomy patients compared to control group patients with total gastrectomy. However, no significant effects were observed in distal gastrectomy patients. They also noted challenges in compliance with ONS due to digestive symptoms like nausea and appetite loss, particularly in total gastrectomy patients [20]. Miyazaki et al. [21] also highlighted that ONS, which contained nutrients such as protein, carbohydrates, fat, vitamins, and minerals (400 kcal/day), significantly reduced BWL in the short term, with the intervention group showing less weight loss at 3 and 6 months. However, the difference was no longer significant at one year, highlighting the importance of adherence to ONS for sustained benefits [21].

### Nutritional Counselling

Two studies in this review explored various forms of nutritional counselling, focusing on individualised, continuous support to optimise patient outcomes following gastrectomy. The study by Takata et al. [22] in Japan implemented nutritional counselling through six sessions, providing personalised dietary advice based on the recovery stage, self-monitoring of weight and food intake, and ONS recommendations when needed. The intervention, which included a low-carbohydrate, high-protein, high-fat diet, significantly reduced BWL and Skeletal Muscle Loss (SML) at 12 months post-gastrectomy compared to the control group [22].

Klassen et al. [23] provided perioperative nutritional counselling, which started before surgery and extended into the postoperative recovery phase. Patients received individualised counselling, including preoperative nutritional assessments, postoperative dietary guidance, and strategies for managing gastrointestinal symptoms. The counselling was adjusted based on the patient’s nutritional risk and recovery progress, with continuous monitoring of food intake and weight. No significant difference was observed between the control and intervention groups in BMI, complications, length of hospital stay, and mortality [23].

### Dietary Education

One study focused on educational or simplified dietary educational intervention. The study from South Korea by Ryu et al. [24] provided simplified dietary education during routine outpatient visits, focusing on general principles of nutrition post-gastrectomy. Patients were given guidance on portion sizes, meal frequency, and avoiding foods that could trigger gastrointestinal discomfort, such as those high in sugar or fat. They reported contradictory findings, where no significant differences were observed in BWL and BMI among their treatment groups, i.e., the non-educated group, after a subtotal gastrectomy [24].

### Nutritional Counselling with ONS

Two studies provided nutritional counselling and ONS as interventions. The study by Meng et al. [12] incorporated nutritional counselling as a post-discharge strategy, involving biweekly counselling, dietary advice, and ONS (500 mL/day) containing high-quality protein, probiotics, and calcium. Patients received counselling on balanced nutrition with specific caloric and protein targets, and adjustments were made based on weight and intake. The intervention improved nutritional outcomes, such as higher BMI, increased caloric and protein intake, reduced BWL, and lowered the incidence of sarcopenia, thereby enhancing chemotherapy tolerance by reducing treatment delays and dose modifications (Table 3) [12].

The study by Yan et al. [25] in China provided nutritional counselling focusing on individualised dietary counselling tailored to each patient’s caloric and protein requirements post-gastrectomy. This included a regular reassessment of the patient’s nutritional intake and adjustment of dietary plans, reporting a significant reduction in BMI loss and higher adherence to energy and protein intake goals among the intervention group compared to controls [25].

### Nutritional Counselling and Education

In the study from South Korea by Kim et al. [11], dietary education was provided to the intervention group, where patients were educated about the importance of maintaining proper nutritional intake to aid recovery post-gastrectomy. This included advice on small, frequent meals, high-protein and high-calorie foods, and strategies for managing common post-gastrectomy symptoms like dumping syndrome and early satiety. This study reported no significant differences in physical factors such as weight and BMI of patients in the intervention group. However, improvements were observed in the functional and behavioural factors [11].

The study by Xie et al. [26] from China included individualised dietary advice to ensure patients consumed enough calories and protein to maintain weight and muscle mass during recovery. The intervention group was provided with educational intervention on the side effects of chemotherapy, in addition to guidance on a diet relevant to their condition, especially post-chemotherapy, and reported significant improvement in calorie and iron intake within a day. Improvements were recorded in the patient’s weight, haemoglobin, total serum protein, and albumin levels in the intervention group compared with the control group during their 21-week intervention [26].

Jiang et al. [27] conducted a 12-week feasibility study in China using a mobile app-based individualised nutritional counselling for participants post gastrectomy. This approach offered personalised dietary advice, weekly meal plans, symptom management strategies and educational resources such as videos and articles tailored to the recovery stage, on nutritional intake using food and ONS. The intervention supported patients in meeting daily energy and protein requirements, with ONS recommended when intake was insufficient. Significant improvements were observed in nutritional behaviour, energy intake, and adherence to protein and energy goals [27].

### ONS, Nutritional Counselling and Physical Activity

The study by Nishida et al. [28] included ONS, nutritional counselling, and physical activity interventions, such as walking 5,000 steps, calf raises, and squats, as part of their study. The authors reported that BCAA-rich ONS, combined with postoperative exercise, reduced the decline in SMI, thereby reducing skeletal muscle loss, improving body composition recovery, and improving QoL shortly after gastrectomy among patients aged 65 and above [28].

### Body Composition Change

In this review, most studies reported favourable body composition changes such as reduced BWL, less SML and reduced loss of SMI. Six studies directly reported BWL in terms of percentage as an outcome [18–22, 24], and in four studies, the percentage BWL was calculated based on body weight measurements at baseline and end-line [11, 25–27]. In three studies, either the baseline or end-line values of body weight were not available [12, 23, 28].

**Fig. 3:**
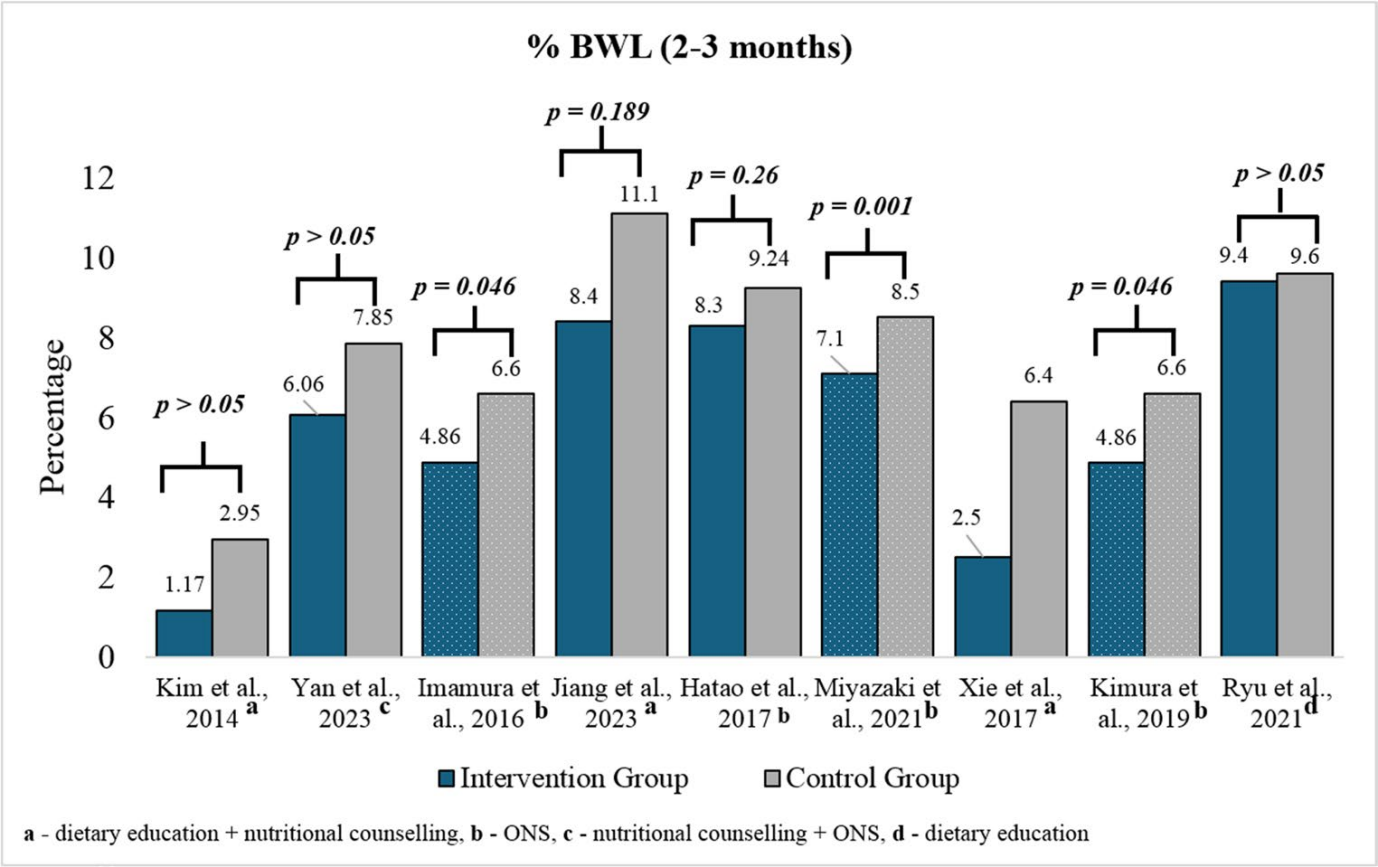
Comparison of BWL among intervention and control groups within 2–3 months post-treatment

The studies show a consistent pattern wherein control groups experienced greater weight loss than intervention groups. Fig. 3 clearly shows the differences in BWL among intervention and control group participants, indicating that interventions consistently demonstrate a significant advantage over control groups in mitigating body weight loss.

**Fig. 4:**
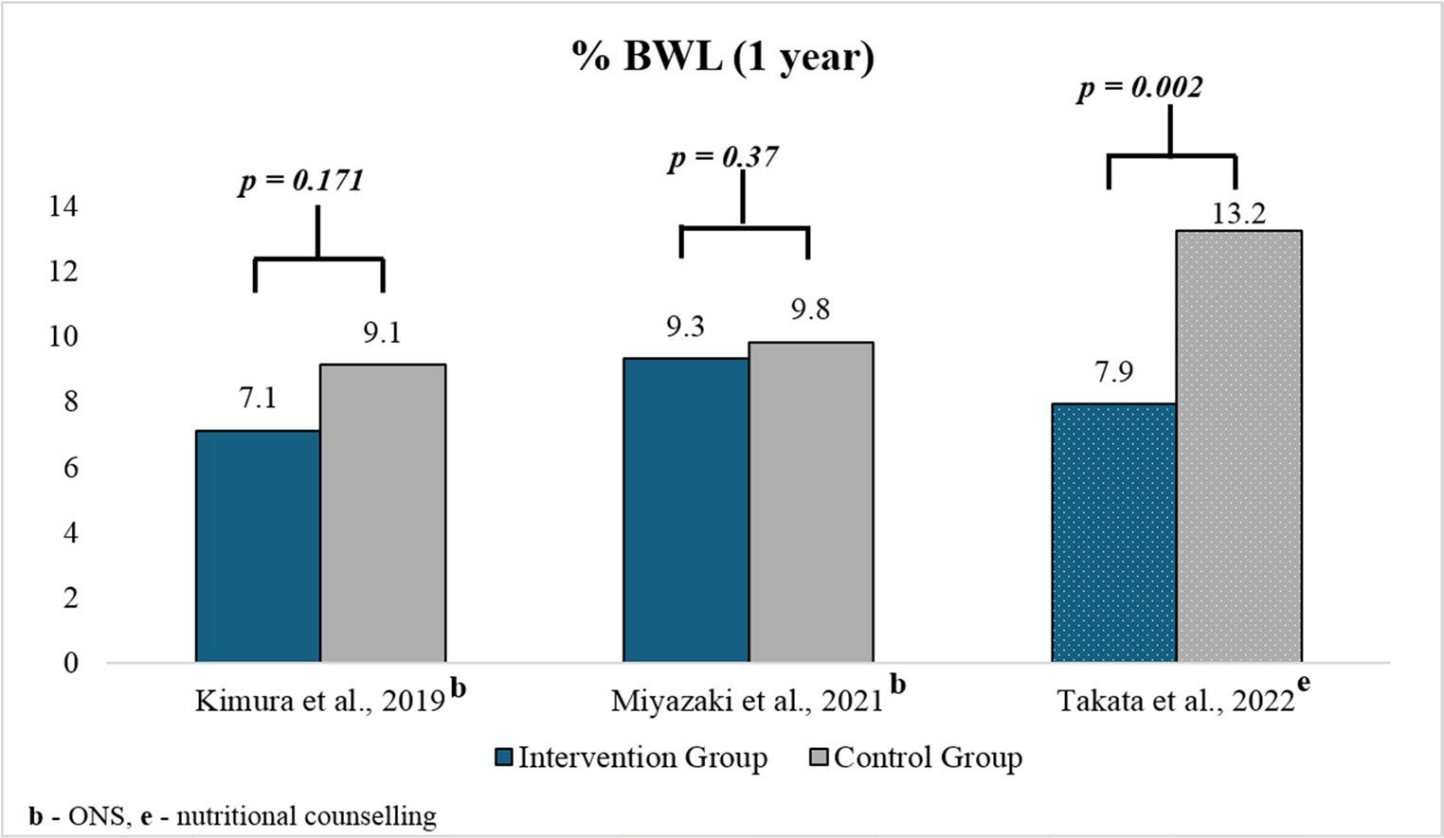
Comparison of BWL among intervention and control groups 12 months post-treatment

The results also showed variations by the time point of intervention. This pattern continued at the 1-year follow-up, where the control groups experienced greater weight loss than. Miyazaki et al. [21] reported the effectiveness of ONS on BWL in the short term, with the ONS group showing less weight loss at 3 months (7.1% vs. 8.5%, *p* = 0.0011) and 6 months. However, at 1 year, the difference was not statistically significant [21]. Similarly, the study by Kimura [19] also reported that the % BWL in 2 months post-surgery was statistically significant; however, it became non-significant one year after surgery **(**Fig. 4**)**. The most pronounced statistically significant difference was observed in Takata et al. [22], with the control group achieving a 13.2% BWL compared to 7.9% in the intervention arm.

Several studies in this review also included SML and SMI as a body composition measure post-gastrectomy. Takata et al. [22] reported that the percentage of SML was significantly lower in the nutritional counselling group compared to the control group at 12 months post-surgery (5.3% vs. 12.8%, *p* = 0.002) [22]. Similarly, Meng et al. [12] measured SMI three months after the intervention and found that patients receiving ONS had a significantly higher SMI than those in the control group (*p* < 0.05) [12]. Nishida et al. [28] also observed dynamic changes in SMI post-surgery, with a 4.6% decrease in the first week followed by a 2.1% increase in the first month, indicating a partial recovery of muscle mass over time [28]. In contrast, Hatao et al. [20] measured skeletal muscle mass but found no significant difference in muscle loss between the ONS and control groups after distal or total gastrectomy (*p* = 0.18). However, they also highlighted that losses of both skeletal muscle and body fat were significantly greater after total gastrectomy compared to distal gastrectomy, particularly among patients without nutritional intervention [20].

#### Effectiveness of Interventions by Type of Gastrectomy

Only four studies specifically reported the effectiveness of nutritional interventions (all using ONS) in patients who underwent different types of gastrectomy, including total and partial (distal and proximal) procedures [18–21].

Imamura et al. [18] reported that within two months post-surgery, the ONS significantly reduced BWL in the intervention group of TG patients compared to the control group of TG patients (5% vs. 9%, *p* = 0.012). While for patients with DG, the results were not significant between the intervention and control groups (4.7% vs. 5.2%, *p* = 0.596) [18]. The extension of the same study for 12 months post-surgery by Kimura et al. [19] reported that ONS were significantly more effective in patients who underwent TG than in those who underwent DG. In TG patients, ONS reduced long-term BWL at 1 year (9.66% vs. 15.11%, *p* = 0.015). However, in DG patients, there was no significant difference in BWL between the ONS and control groups (5.81% vs. 6.0%, *p* = 0.933) [19].

Hatao et al. [20] also reported similar findings, showing that ONS significantly reduced weight loss in TG patients, with the ONS group retaining 88.5% of their preoperative body weight compared to 85.6% in the control group (*p* < 0.05). However, for distal gastrectomy patients, no significant differences in body weight or skeletal muscle mass were observed between the intervention and control groups, highlighting that distal gastrectomy patients may not derive as much benefit from ONS alone [20]. Although Miyazaki et al. [21] did not provide specific breakdowns of BWL by type of gastrectomy at all time points, the study indicated that compliance with ONS played a critical role, especially for TG patients who are more vulnerable to severe nutritional deficits [21]. (Supplementary Table 3)

However, Ryu et al. [24] focused exclusively on patients who underwent subtotal (partial) gastrectomy and reported that simplified dietary education alone was ineffective as a nutritional intervention following gastrectomy [24].

#### Quality of Life

Six studies assessed QoL outcomes (Table 4). However, the magnitude and direction of improvements differed; only one reported a statistically significant between-group improvement, while the others demonstrated non-significant or unclear differences. Most improvements were within-group trends, and two studies did not report statistical testing. Kim et al. (2014) reported a significantly greater improvement in Functional Assessment of Cancer Therapy - General (FACT-G) scores in the intervention group compared with controls at 12 weeks (*p* < 0.001) [11].

**Table 4:** Quality of life reported in studies included in the review

Study title	QoL tool	Intervention	Interval	Intervention group	Control group	Significance
Nishida et al., [28]	EQ-5D	ONS, nutritional counselling, and physical actitivty	T0 (0 week)	0.89	-	NR
			T1 (1 week)	0.88	-	
			T2 (4 week)	0.83	-	
Kim et al., [11]	FACT-G	Nutritional education, nutritional counselling	T0 (0 week)	61.92	58.09	*p* < 0.001
			T1 (12 week)	86.27	60.09	
Meng et al., [12]	EORTC-QLQ C-30	ONS, nutrtitional counselling	T0 (0 week)	-	-	*p* = 0.256^a^
			T1 (12 week)	75.00	73.00	
Jiang et al., [27]	EORTC-QLQ C-30	Nutritional education, nutritional counselling	T0 (0 week)	80.48	71.09	
			T1 (4 week)	77.26	65.11	*p* = 0.609^a^
			T2 (12 week)	75.66	67.05	
Yan et al., [25]	EORTC-QLQ C-30	Nutritional counselling, ONS	T0 (0 week)	-	-	NR
			T1 (12 week)	75.00	66.00	
			T1 (8 week)	71.90	65.10	
Hatao et al., [20]^b^	EORTC-QLQ C-30	ONS	T0 (0 week)	67.00	67.00	NR
			T1 (12 week)	67.00	63.00	

*NR – not relevant; a - Group-by-Time Interaction Effects; b – QoL of patients with total gastrectomy*

In contrast, the remaining five studies reported either within-group improvements or non-significant between-group differences. Meng et al. [12] observed similar improvements in EORTC-QLQ-C30 scores in both the intervention and control groups, with no significant difference between the arms (*p* = 0.26) [12]. Jiang et al. [27] also found higher QoL scores in the intervention group at multiple time points; however, the differences were not statistically significant (*p* = 0.61) [27]. Yan et al. [25] reported higher QoL scores in the intervention group at 12 weeks, although significance was not reported [25]. Nishida et al. [28], which lacked a control group, reported a slight decline in EQ-5D scores over four weeks; however, statistical significance was not reported [28]. Similarly, Hatao et al. [20] reported no clear difference between intervention and control groups, and significance was not reported [20].

## Discussion

This is the first comprehensive systematic review that examined the effects of various nutritional interventions, including ONS, nutritional counselling and dietary education, on postoperative outcomes such as body composition and QoL of individuals with gastrectomy. Interventions, particularly ONS and nutritional counselling with regular monitoring, led to a significant reduction in BWL and SML [18–21]. Improvements in QoL were statistically significant in only one study; however, several studies noted positive, albeit non-significant, changes—especially with combined nutritional counselling and education [11]. The degree of benefit varied depending on the intensity and type of intervention. Studies with personalised and continuous nutritional counselling showed more robust improvements across all outcomes compared to those employing simplified or one-time educational approaches.

Nutritional counselling involves a two-way interaction between patients and the service provider, i.e. dietician, nutritionist or the medical team, in which the nutritional needs of the patients are discussed, maintained and monitored on a regular basis [29]. This personalised approach has been shown to be critical in reducing BWL and preserving muscle mass. When implemented through consistent follow-up sessions, personalised dietary advice, and active patient engagement, it significantly reduces BWL and SML while improving compliance with dietary recommendations. The findings of this review align with earlier studies suggesting that sustained, individualised dietary counselling significantly improves nutritional outcomes and patient compliance [30, 31]. Similar positive outcomes have been observed in colorectal cancer patients receiving such interventions [32]. Additionally, evidence from another study involving both stomach and colon cancer patients highlights the beneficial impact of nutritional counselling on postoperative quality of life [33].

ONS are multi-nutrient products and are available either in the liquid, semi-solid or powder form [34]. The findings of this review align closely with existing literature on the effectiveness of nutritional counselling and ONS in improving postoperative outcomes for gastric cancer patients. When combined with nutritional counselling, ONS consistently demonstrated benefits in improving body composition or reducing sarcopenia. Studies found significant reductions in BWL among patients who received ONS post-gastrectomy [18, 19]. Another study on head and neck cancer patients found that when patients received ONS and nutritional counselling, they effectively maintained body weight and had improved QoL and better postoperative treatment tolerance [35].

In the systematic review by Lee et al. (2016), studies were analysed separately based on types of interventions such as nutritional counselling alone, nutritional counselling with ONS, and ONS without nutritional counselling. They found that nutritional counselling, whether combined with ONS or not, led to improved nutritional outcomes, while ONS alone did not. Although the included studies varied widely in cancer types, stages, intervention durations, control group comparisons, and treatments (radiotherapy, chemotherapy, or surgery), the positive impact of nutritional counselling on nutritional status remained consistent [36].

The findings of this review also reinforce that dietary education alone, even when extended over the long term, is insufficient in improving nutritional outcomes among post-gastrectomy patients [24]. In contrast, interventions that incorporated personalised education and tailored nutritional counselling demonstrated notable improvements in patients’ nutritional status [26]. Furthermore, combining dietary education with counselling has also been associated with enhanced quality of life outcomes [11]. These results highlight the importance of moving beyond conventional education towards more comprehensive strategies integrating interventions such as individualised nutritional counselling and, where appropriate, ONS [24]. By providing tailored nutritional guidance alongside educational support, patients are better equipped to comply with their treatment plans and manage the side effects associated with chemotherapy [26]. Incorporating patients’ perspectives into dietary interventions leads to improved outcomes and higher-quality care [11].

While most studies in this review show improvements in body composition following nutritional interventions, interpreting these results is challenging, as not all studies reported the outcomes by different types of gastrectomies, such as total, distal, and proximal. Research consistently indicates that patients undergoing total gastrectomy face a higher risk of severe weight loss and nutritional deficiencies, making interventions for weight maintenance and muscle preservation more critical compared to those undergoing distal or proximal gastrectomy [37–39]. Only a limited number of studies in this review specifically compared outcomes between patients of total and partial gastrectomies (distal and proximal), demonstrating that patients with total gastrectomy benefited more from ONS in terms of reducing BWL [18–20]. The fact that interventions were particularly effective for patients with total gastrectomy may be due to their heightened nutritional needs. Although other studies indicated the overall benefits of nutritional interventions, they did not specify results based on the type of gastrectomy. This lack of specificity in these interventional studies limits a comprehensive understanding of how different surgical approaches influence the effectiveness of nutritional interventions [11, 25, 26]. Although some studies have demonstrated the effectiveness of interventions in patients with total gastrectomy, there is a notable lack of research specifically focused on this patient subgroup. This raises the need for more detailed research, differentiating outcomes by type of gastrectomy.

Nutritional counselling, especially when paired with ONS, should be integrated into standard postoperative care to reduce the risk of SML, malnutrition, and BWL. Using digital platforms to deliver nutritional counselling presents a promising avenue for expanding access to personalised care, especially in resource-limited settings [40, 41]. Furthermore, the findings suggest that simplified dietary education alone may not yield meaningful improvements, underscoring the need for more intensive, follow-up-based interventions.

In cancer care, maintaining an appropriate body weight is crucial, as both undernutrition and obesity can adversely affect treatment outcomes and overall patient health [42, 43]. Weight loss is a common issue among patients with gastrectomy, often worsened by treatment-related side effects, making nutritional support essential. However, the role of baseline body composition measures, such as BMI, muscle mass, body fat, bone density, etc., in guiding dietary interventions is frequently overlooked in research. Interpreting the impact of nutritional interventions on weight loss and body composition is complex because weight loss includes various components, such as muscle mass, body fat, bone density, and hydration, which are rarely distinguished in studies. The loss of muscle mass can severely impair physical function, especially in underweight patients, compared to fat loss, which might be less detrimental or even beneficial in those with obesity. Thus, the implications of weight loss vary greatly depending on an individual’s baseline weight status, with underweight individuals facing more severe adverse effects. Additionally, this oversight might stem from the geographic context of the studies, as many are conducted in regions where obesity is not a prevalent concern, leading to a focus on preventing weight loss rather than addressing the broader aspects of body composition changes.

Notably, 12 of the 13 studies reviewed were conducted in East Asian countries, where obesity is less prevalent among the general population. Moreover, in the studies that did report baseline BMI, the values were less than 25 kg/m², indicating a leaner population, except for one study from Germany, where the baseline BMI was around 26.7 kg/m² [23]. Due to these factors, it can be argued that the interventions were more likely focused on maintaining body weight/mass rather than addressing obesity. This might explain these studies’ limited discussion on body composition measures, such as BMI, fat loss, etc. In regions where obesity or overweight is more prevalent in the population, interventions may focus more on excess weight rather than only addressing weight maintenance, highlighting the importance of considering multiple baseline body composition measures in designing and evaluating dietary interventions.

The results of this review have several important clinical implications for the management of gastric cancer patients post-gastrectomy. The lack of statistically significant results in outcomes may be attributed to limited sample sizes or other methodological constraints, which could reduce the power to detect meaningful associations. However, the effect sizes observed in these studies should not be overlooked. Even in the absence of statistical significance, consistent differences between control and intervention groups suggest potentially meaningful trends that need further investigation. These findings underscore not only the effectiveness of nutritional interventions but also the critical role of trained professionals, particularly dietitians, in delivering them. In many low- and middle-income countries (LMICs), however, the nutritional workforce remains under-resourced and undervalued [44]. Patients often rely solely on clinicians or surgical oncologists for dietary advice, despite the complexity of nutritional needs following gastrectomy [45]. The limited integration of dietitians into routine oncology care reflects broader systemic gaps, including inadequate training, staffing, and recognition of their role within multidisciplinary teams [46–48].

### Strengths and Limitations

The strength of this review is its focus on randomised controlled trials and multicenter studies, which provide high-quality evidence on the effectiveness of nutritional interventions. However, several limitations should be considered. First, the heterogeneity in study design, intervention components, and follow-up duration may have contributed to the variability in findings across studies. Second, most studies had relatively small sample sizes, which may have limited the statistical power to detect significant between-group differences in outcomes such as QoL and skeletal muscle mass. Third, 12 of the 13 studies were conducted in East Asia, which restricts the generalizability of the findings to broader global settings where patient profiles, dietary patterns, and healthcare systems differ. Finally, the limited availability of long-term follow-up data constrains our understanding of the durability of nutritional benefits over time.

## Conclusion

In conclusion, this systematic review highlights the important role of nutritional counselling and ONS in improving postoperative outcomes for gastric cancer patients. Among the various approaches, ONS, personalised nutritional counselling, and nutritional education consistently led to better outcomes in reducing BWL, preserving SML and improving QoL. The heterogeneity in reporting and lack of standardisation in body composition outcomes limit comparability, yet trends indicate that early, continuous, and individualised support is essential for optimal recovery. The findings advocate for the integration of nutritional counselling, ONS and nutritional education into standard postoperative care, supported by digital delivery methods to expand reach.

While the overall conclusion is that interventions are effective, this highlights the crucial role of dietitians, especially in low and middle-income settings where people often rely on clinicians and gastroenterologists. In many LMICs, dietitians are not routinely integrated into oncology care pathways. Ensuring that patients have access to dietary guidance from a professional can help address these diverse needs effectively. Future research should prioritise stratification by gastrectomy type and incorporate uniform baseline and endline body composition assessments to better tailor interventions and interpret outcomes across diverse populations.

## Supplementary Information

Below is the link to the electronic supplementary material.

Supplementary File 1 (DOCX 29.8 KB)

## Data Availability

All data, analytic methods, and study materials will be made available to other researchers upon request.

## Declarations

### Data transparency statement

All data, analytic methods, and study materials will be made available to other researchers upon request.

## Abbreviations

PRISMA: Preferred Reporting Items for Systematic Reviews and Meta-Analysis
RCT: Randomised controlled trials
JBI: Joanna Briggs Institute
BCAA: Branched-chain amino acid
ED: Elemental Diet
ONS: Oral Nutritional Supplement
BWL: Body Weight Loss
PPDI: Patient participation-based dietary intervention
QoL: Quality of Life
IG: Intervention Group
CG: Control Group
GC: Gastric cancer
BMI: Body Mass Index
SMI: Skeletal Mass Index
SML: Skeletal Muscle Loss
TG: Total Gastrectomy
EQ: 5D-EuroQol 5 Dimension
FACT-G: Functional Assessment of Cancer Therapy-General
EORTC: QOL-European Organization for the Research and Treatment of Cancer Quality of Life Questionnaire
LMICs: Low and Middle Income Countries
