# Supplementary material for: Effect of Dietary Interventions on Body Composition and Quality of Life in Stomach Cancer Survivors after Gastrectomy: A Systematic Review
Source: J Gastrointest Cancer. 2026 Jan 13;57(1):11. doi: 10.1007/s12029-025-01388-5 (PMC12799704; doi:10.1007/s12029-025-01388-5)
Supplement: Supplementary file 1 — (DOCX 29.8 KB) [file 12029_2025_1388_MOESM1_ESM.docx]

**Supplementary tables**

**Supplementary Table 1:** Search string

| Databases | Search string |
| --- | --- |
| MEDLINE/OVID | 1 ("Stomach Neoplasm*" or "Gastric cancer" or "Gastric Malignancy" or "Gastric Neoplasm*" or "Cancer of Stomach" or "Stomach tumour" or "Stomach carcinoma*" or "Gastric tumo*" or "Gastric carcinoma*").mp.  [mp=title, book title, abstract, original title, name of substance word, subject heading word, floating sub-heading word, keyword heading word, organism supplementary concept word, protocol supplementary concept word, rare disease supplementary concept word, unique identifier, synonyms, population supplementary concept word, anatomy supplementary concept word]  2 (“Gastrectomy" or "Gastrectomies" or "Total gastrectomy" or "Subtotal gastrectomy" or "Sleeve gastrectomy" or "Partial gastrectomy").mp. [mp=title, book title, abstract, original title, name of substance word, subject heading word, floating sub-heading word, keyword heading word, organism supplementary concept word, protocol supplementary concept word, rare disease supplementary concept word, unique identifier, synonyms, population supplementary concept word, anatomy supplementary concept word]  3 Diet Therapy/ ("Diet Therapy" or "Dietary Supplements" or "Diet" or "Dietary intervention" or "Dietary intake" or "Diet Therapies" or "Dietary Modification" or "Dietary Supplements" or "Dietary Restriction" or "Diet change" or "Healthy eating" or "Healthy nutrition" or "Dietary patterns" or "Whole diets" or "Nutrition" or "Nutritional supplements" or "Nutritional advice" or "Nutritional counselling" or "Nutritional support" or "Nutritional intervention" or "Food based interventions" or "Food interventions" or "Food recommendations" or "Diet* behavio*").mp. [mp=title, book title, abstract, original title, name of substance word, subject heading word, floating sub-heading word, keyword heading word, organism supplementary concept word, protocol supplementary concept word, rare disease supplementary concept word, unique identifier, synonyms, population supplementary concept word, anatomy supplementary concept word]  4 Clinical Trial/ Pilot*/ Feasibility*/ ("Clinical trial" or “Pilot*” or “Feasibility*”).mp. [mp=title, book title, abstract, original title, name of substance word, subject heading word, floating sub-heading word, keyword heading word, organism supplementary concept word, protocol supplementary concept word, rare disease supplementary concept word, unique identifier, synonyms, population supplementary concept word, anatomy supplementary concept word]  5 Stomach Neoplasms/  6 Gastrectomy/  7 Diet Therapy/  8 Clinical Trial/  9 Pilot/  10 Feasibility studies/  11 1 or 5  12 2 or 6  13 3 or 7  14 4 or 8 or 9 or 10  15 11 and 12 and 13 and 14  16 limit 15 to (english language and humans and yr="2000 -Current") |
| EMBASE/OVID | 1 ("Stomach Neoplasm*" or "Gastric cancer" or "Gastric Malignancy" or "Gastric Neoplasm*" or "Cancer of Stomach" or "Stomach tumour" or "Stomach carcinoma*" or "Gastric tumo*" or "Gastric carcinoma*").mp. [mp=title, abstract, heading word, drug trade name, original title, device manufacturer, drug manufacturer, device trade name, keyword heading word, floating subheading word, candidate term word]  2 ("Gastrectomy" or "Gastrectomies" or "Total gastrectomy" or "Subtotal gastrectomy" or "Sleeve gastrectomy" or "Partial gastrectomy").mp. [mp=title, abstract, heading word, drug trade name, original title, device manufacturer, drug manufacturer, device trade name, keyword heading word, floating subheading word, candidate term word]  3 ("Diet Therapy" or "Dietary Supplements" or "Diet" or "Dietary intervention" or "Dietary intake" or "Diet Therapies" or "Dietary Modification" or "Dietary Supplements" or "Dietary Restriction" or "Diet change" or "Healthy eating" or "Healthy nutrition" or "Dietary patterns" or "Whole diets" or "Nutrition" or "Nutritional supplements" or "Nutritional advice" or "Nutritional counselling" or "Nutritional support" or "Nutritional intervention" or "Food based interventions" or "Food interventions" or "Food recommendations" or "Diet* behavio*").mp. [mp=title, abstract, heading word, drug trade name, original title, device manufacturer, drug manufacturer, device trade name, keyword heading word, floating subheading word, candidate term word]  4 ("Clinical trial" or “Pilot*” or “Feasibility*”).mp. [mp=title, abstract, heading word, drug trade name, original title, device manufacturer, drug manufacturer, device trade name, keyword heading word, floating subheading word, candidate term word]  5 Stomach tumor/  6 Gastrectomy/  7 Diet Therapy/  8 Clinical Trial/  9 Pilot Study/  10 Feasibility study/  11 1 or 5  12 2 or 6  13 3 or 7  14 4 or 8 or 9 or 10  15 11 and 12 and 13 and 14  16 limit 15 to (english language and humans and yr="2000 -Current") |
| Cochrane library | ((Stomach Neoplasms) OR (Gastric cancer) OR (Gastric Malignancy) OR (Gastric Neoplasms) OR (Cancer of Stomach) OR (Gastric tumour) OR (Gastric carcinoma) OR (Stomach tumour) OR (Stomach carcinoma)) AND ((Gastrectomy) OR (Gastrectomies) OR (Total gastrectomy) OR (Subtotal gastrectomy) OR (Sleeve gastrectomy) OR (Partial gastrectomy)) AND ((Diet Therapy) OR (Diet) OR (Dietary intervention) OR (Dietary intake) OR (Diet Therapies) OR (Dietary Modification) OR (Dietary Restriction) OR (Diet change) OR (Diet behavio*) OR (Dietary counselling) OR (Healthy eating) OR (Healthy nutrition) OR (Dietary patterns) OR (Whole diets) OR (Nutrition) OR (Nutritional advice) OR (Nutritional counselling) OR (Nutritional support) OR (Nutritional intervention) OR (Food based interventions) OR (Food interventions) OR (Food recommendations)) AND ((Clinical trial*) OR (Feasibility*) OR (Pilot*)) |
| Web of Science | **(((((((((ALL=(“Stomach neoplasm*”)) OR ALL=(“Gastric cancer”)) OR ALL=(“Gastric Malignancy”)) OR ALL=(“Gastric Neoplasm*”)) OR ALL=(“Cancer of Stomach” )) OR ALL= (“Gastric tumo*")) OR ALL = (“Gastric carcinoma*”)) OR ALL = ("Stomach tumo*")) OR ALL= ("Stomach carcinoma*") )AND ((((((ALL=(“Gastrectomy”)) OR ALL=(“Gastrectomies”)) OR ALL=(“Total gastrectomy”)) OR ALL=(“Subtotal gastrectomy”)) OR ALL=(“Sleeve gastrectomy”)) OR ALL=(“Partial gastrectomy”)) AND (((((((((((((((((((((ALL=(“Diet” )) OR ALL=(“Dietary intervention”)) OR ALL=(“Dietary intake”)) OR ALL=(“Diet Therapies”)) OR ALL=(“Dietary Modification”)) OR ALL=(“Dietary Restriction”)) OR ALL=(“Diet change”)) OR ALL=(“Healthy eating”)) OR ALL=(“Healthy nutrition”)) OR ALL=(“Dietary patterns”)) OR ALL= (“Dietary counselling”)) OR ALL=(“Whole diets”)) OR ALL=(“Nutrition”)) OR ALL=(“Nutritional advice”)) OR ALL=(“Nutritional counselling”)) OR ALL=(“Nutritional support”)) OR ALL=(“Nutritional intervention”)) OR ALL=(“Food based interventions”)) OR ALL=(“Food interventions”)) OR ALL=(“Food recommendations”)) OR ALL = (“Diet* behavio*”)) AND (((ALL = (Clinical trial*)) OR ALL= (“Feasibility*”)) OR ALL= (“Pilot”))** |
| ProQuest | **((“Stomach neoplasms”) OR (“Gastric cancer”) OR (“Gastric Malignancy”) OR (“Gastric Neoplasms”) OR (“Cancer of Stomach” ) OR (“Gastric tumo*") OR (“Gastric carcinoma*”) OR ("Stomach tumo*") OR ("Stomach carcinoma*" )) AND ((“Gastrectomy”) OR (“Gastrectomies”) OR (“Total gastrectomy”) OR (“Subtotal gastrectomy”) OR (“Sleeve gastrectomy”) OR (“Partial gastrectomy”)) AND ((“Diet Therapy”) OR (“Diet”) OR (“Dietary intervention”) OR (“Dietary intake”) OR (“Diet Therapies”) OR (“Dietary Modification”) OR (“Dietary Restriction”) OR (“Diet change”) OR (“Diet* behavio*”) OR (“Dietary counselling”) OR (“Healthy eating”) OR (“Healthy nutrition”) OR (“Dietary patterns”) OR (“Whole diets”) OR (“Nutrition”) OR (“Nutritional advice”) OR (“Nutritional counselling”) OR (“Nutritional support”) OR (“Nutritional intervention”) OR (“Food based interventions”) OR (“Food interventions”) OR (“Food recommendations”)) AND ((“Clinical trial*”) OR (Feasibility*) OR (Pilot*))** |
| CINAHL | S1 - (TX gastrectomy OR TX total gastrectomy OR TX subtotal gastrectomy OR TX sleeve gastrectomy OR TX partial gastrectomy)  S2 - (TX stomach neoplasms OR TX gastric cancer OR TX gastric malignancy OR TX gastric neoplasm OR TX cancer of stomach OR TX gastric tumour OR TX gastric carcinoma OR TX stomach tumour OR TX stomach carcinoma)  S3 - (TX diet OR TX dietary intervention OR TX dietary intake OR TX diet therapy [mesh] OR TX dietary modifications OR TX dietary restrictions OR TX diet change OR TX diet behavior OR TX dietary counseling OR TX healthy eating OR TX healthy nutrition OR TX dietary patterns OR TX whole diets OR TX nutrition OR TX nutritional advice OR TX nutritional counseling OR TX nutritional support OR TX nutritional intervention OR TX food based interventions OR TX food intervention OR TX food recommendation)  S4 - (TX clinical trial OR TX feasibility OR TX pilot)  **S5 - (S1 AND S2 AND S3 AND S4)**  Limiters - Published Date: 20000101-; English Language; Human |

**Supplementary Table 2:** Study Characteristics

| **Author, Year** | **Country** | **Study design** | **Specific Interventions** | **Common Interventions for Both Groups** | **Baseline characteristics** | **Endline characteristics** | **Outcome** | **Remarks** |
| --- | --- | --- | --- | --- | --- | --- | --- | --- |
| Kim et al., 2014 | South Korea | RCT | Education, Nutritional counselling | Group nutritional education, nutritional education by a dietitian, and general discharge education | **BMI**  **IG** - 22.20 ± 3.69  **CG** - 21.98 ± 2.45  **BWT**  **IG -** 58.89 ± 10.78  **CG** - 58.15 ± 8.46 | **BMI**  **IG** - 21.98 ± 3.71  **CG** - 21.29 ± 2.29  **BWT**  **IG -** 58.20 ± 10.62  **CG** - 56.43 ± 88.43 | Weight, height, triceps, BMI,  and scapular muscle mass and quality of life. |  |
| Imamura et al., 2016  18/06/25 2:22:00 AM | Japan | RCT | ONS, (oral elemental diet, Elental, 300 kcal/day) | Regular diet | **BMI**  **IG** - 21.9 ± 3.1  **CG** - 22.4 ± 3.4  **BWT**  **IG -** 57.5 ± 10.8  **CG -** 58.7 ± 12.0 | **BMI**  NR  **BWT**  NR | BWL, nutrition‐related blood parameters. |  |
| Xie et al., 2017  18/06/25 2:22:00 AM | China | RCT | Intensive individualised nutritional & educational intervention | - | **BMI**  NR  **BWT**  **IG -** 53.75 ± 7.62  **CG -** 53.48 ± 9.15 | **BMI**  NR  **BWT**  **IG -** 51.84 ± 7.24  **CG -** 49.28± 8.98 | Weight, hemoglobin, total serum protein, and albumin |  |
| Hatao et al., 2017 | Japan, Taiwan | RCT | ONS (concenttated liquid diet ANOM) | Usual postoperative diet | **BMI**  **IG -** NR **CG** - NR  **BWT**  **IG** - 61.2 ± 11.0 **CG** - 59.5 ± 10.7 | **BMI**  **IG -** NR **CG** - NR  **BWT**  **IG** - 55.9 ± 10.0 **CG** - 54.0 ± 9.2 | Weight and body compositions (fat and skeletal muscle), QoL, blood chemistry data |  |
| Kimura et al., 2019 | Japan | RCT | ONS, (oral elemental diet, Elental, 300 kcal/day) | Regular diet | **BMI**  NR **BWT**  **IG** - 57.5 ± 10.9  **CG** - 58.6 ± 12.5 | **BMI**  NR  **BWT**  NR | BWL, nutrition‐related blood parameters. |  |
| Meng et al., 2021 | China | RCT | ONS, nutrtitional counselling (dietary advice and an  oral intake of Nutren® Optimum at a 500 mL/day) | Dietary advice | **BMI**  NR  **BWT**  NR | **BMI**  NR  **BWT**  NR | Weight, BMI, SMI, sarcopenia prevalence, and QoL. |  |
| Ryu et al., 2021 | South Korea | Case-control | Dietary education | - | **BMI**  **IG** – 24.5 ± 2.7  **CG** – 24.3 ± 2.8  **BWT**  **IG** – 66.2 ± 10.0  **CG** - 65.9 ± 10.4 | **BMI**  NR  **BWT**  NR | Body weight, BMI, absolute lymphocyte count (ALC), serum albumin level, and PNI | Prospectively identified retrospectively analysed |
| Miyazaki et al., 2021 | Japan | RCT | ONS, (Racol NF 400 kcal/day) | Regular diet | **BMI**  **IG** - 22.5 ± 3.2 **CG**- 22.6 ± 3.2  **BWT**  **IG** - 59.2 ± 11.2 **CG** - 59.4 ± 11.3 | **BMI**  **IG** -  **CG**-   **BWT**  **IG** -  **CG** - | Body weight, BWL, nutrition-related and other blood laboratory data |  |
| Takata et al., 2022 | Japan | Case-control | Perioperative and post-discharge personalised comprehensive nutritional counselling. | Pre-discharge nutritional counselling | **BMI**  **IG** – 22.9 ± 3.6  **CG** – 21.7 ± 3.2  **BWT**  **IG –** 58.6 ± 12.4  **CG –** 60.6 ± 12.1 | **BMI**  NR  **BWT**  NR | BWL, SML, and nutrition-related blood parameters | Case-control identified retrospectively |
| Klassen et al., 2023 | Germany | Case-control | Preoperative and postoperative nutritional support with counselling  High-caloric fluid supplements (2–3 potions (200 mL) per day with 2.0 kcal/mL) and enteral or parenteral nutrition | Postoperative supportive parenteral nutrition (SMOF lipid at 2200 kcal/d) | **BMI**  **IG** – 26.7 ± 5.6  **BWT**  **IG** – 80.60 ± 19.06 | **BMI**  **IG** – 26.2 ± 5.5  **BWT**  **IG** – 78.86 ± 18.03 | Weight, Nutritional Risk Screening (NRS), phase angle | Pre-post operative |
| Nishida et al., 2023 | Japan | Prospective | ONS, Nuritional coinselling, and physical actitivty  (Organised rehabilitation program, 125mL pack of the protein (BCAA)-rich supplemen,walking squatting and calf-raises.) | - | **BMI**  **Total** – 22.3 ± 3.6  **BWT**  **Total** – 57.9 ± 11.2 | **BMI**  **Total** – 21.0 ± 3.6  **BWT**  **Total** – 54.2 ± 10.3 | SMI, Lean body mass, fat mass, body weight, serum albumin level, hand grip strength, gait speed, and QOL |  |
| Jiang et al., 2023 | China | RCT | Nutritional education, nutritional counselling  (12-week individualised mHealth nutrition intervention, biweekly nutrition consultation) | A food atlas helping estimate the amount of food, nutritional education handbook | **BMI**  **IG** - 22.37 ± 3.43  **CG** - 24.72 ± 2.93  **BWT**  **IG -** 60.52 ± 10.52  **CG -** 66.93 ± 9.85 | **BMI**  **IG -** 20.57 ± 2.81  **CG -** 21.77 ± 2.53  **BWT**  **IG -** 55.43 ± 8.25 **CG -** 59.50 ± 9.84 | Weight; BMI; nutritional status tested using PG-SGA point, nutritional risk and QoL |  |
| Yan et al., 2023 | China | RCT | Nutritional counselling, ONS | Conventional dietary counselling | **BMI**  **IG** – 22.79 ± 2.69  **CG** – 23.59 ± 3.66  **BWT**  **IG** – 64.44 ± 11.12  **CG** – 64.31 ± 12.23 | **BMI**  **IG** – 21.41 ± 2.69  **CG** – 21.67 ± 3.12  **BWT**  **IG** – 60.53 ± 10.70  **CG** – 59.26 ± 11.52 | Body weight, 24-h calorie and protein intake, blood parameters, QoL |  |

*RCT – Randomised control trial, QoL – Quality of Life, ED – Elemental Diet, PPDI -Patient Participation Based Dietary Intervention, BWL – Body weight loss, BMI- Body mass index, SML- Skeletal Muscle Loss, SMI - Skeletal Muscle Index, IG – Intervention group, CG – Control group*

**Supplementary table 3:** BWL of patients by types of gastrectomy and duration of intervention.

| **Studies** | **1-3 Months** | | | | | | **6 months** | | | | | | **1 year** | | | | | |
| --- | --- | --- | --- | --- | --- | --- | --- | --- | --- | --- | --- | --- | --- | --- | --- | --- | --- | --- |
|  | **All** | | **TG** | | **PG/DG** | | **All** | | **TG** | | **PG/DG** | | **All** | | **TG** | | **PG/DG** | |
|  | **CG** | **IG** | **CG** | **IG** | **CG** | **IG** | **CG** | **IG** | **CG** | **IG** | **CG** | **IG** | **CG** | **IG** | **CG** | **IG** | **CG** | **IG** |
| Kimura et al., 2019 | 6.6% | 4.8% | 9.0% | 5.0% | 5.2% | 4.7% | 9.2% | 6.9% | 13.3% | 8.8% | 7.0% | 6.0% | 9.1% | 7.1% | 15.1% | 9.7% | 6.0% | 5.8% |
|  | ***p = 0.046*** | | ***p = 0.012*** | | *p = 0.596* | | ***p = 0.083*** | | ***p = 0.042*** | | *p = 0.493* | | *p = 0.171* | | ***p = 0.015*** | | *p = 0.933* | |
| Miyazaki et al., 2021 | 8.50% | 7.10% | 12.10% | 11.00% | 6.70% | 5.40% | 9.70% | 8.60% | - | - | - | - | 9.80% | 9.30% | - | - | - | - |
|  | ***p = 0.0011*** | | *p = 0.25* | | ***p = 0.0056*** | | ***p = 0.0228*** | | - | - | - | - | *p = 0.37* | | - | - | - | - |
| Imamura | 6.60% | 4.86% | 9.0% | 5.0% | 5.2% | 4.7% | - | - | - | - | - | - | - | - | - | - | - | - |
|  | ***p = 0.046*** | | ***p = 0.012*** | | *p = 0.596* | | - | - | - | - | - | - | - | - | - | - | - | - |
| Hatao et al., 2017 | 9.2% | 8.3% | 14.6% | 11.1% | 6.2% | 6.7% | - | - | - | - | - | - | - | - | - | - | - | - |
|  | *p = 0.26* | | ***p = 0.03*** | | *p = 0.26* | | - | - | - | - | - | - | - | - | - | - | - | - |
| Ryu et al., 2021 | 9.40% | 9.60% | - | - | - | - | 9.70% | 10% | - | - | - | - | 11.10% | 10.40% | - | - | - | - |
|  | *p>0.05* | | - | - | - | - | - | - | - | - | - | - | - | - | - | - | - | - |
| Jiang et al., 2023 | 11.10% | 8.41% | - | - | - | - | - | - | - | - | - | - | - | - | - | - | - | - |
|  | *p = 0.189* | | - | - | - | - | - | - | - | - | - | - | - | - | - | - | - | - |
| Kim et al., 2014 | 2.95% | 1.17% | - | - | - | - | - | - | - | - | - | - | - | - | - | - | - | - |
|  | *p = 0.35* | | - | - | - | - | - | - | - | - | - | - | - | - | - | - | - | - |
| Yan et al., 2023 | 7.85% | 6.06% | - | - | - | - | - | - | - | - | - | - | - | - | - | - | - | - |
|  | *p = 0.118* | | - | - | - | - | - | - | - | - | - | - | - | - | - | - | - | - |
| Xie et al., 2017 | 6.46% | 2.54% | - | - | - | - | 7.85% | 3.55% | - | - | - | - | - | - | - | - | - | - |
|  | - | | - | - | - | - | - | | - | - | - | - | - | - | - | - | - | - |
|  |  |  | - | - | - | - | - | - | - | - | - | - | - | - | - | - | - | - |
| Takata et al., 2022 | - | - | - | - | - | - | 12.30% | 7.80% | - | - | - | - | 13.20% | 7.90% | - | - | - | - |
|  | - | - | - | - | - | - | ***p = 0.001*** | | - | - | - | - | ***p = 0.002*** | | - | - | - | - |
| Klassen et al., 2023 | - | - | - | - | - | - | - | - | - | - | - | - | - | - | - | - | - | - |
|  | - | - | - | - | - | - | - | - | - | - | - | - | - | - | - | - | - | - |
| Meng et al., 2021 | - | - | - | - | - | - | - | - | - | - | - | - | - | - | - | - | - | - |
|  | - | - | - | - | - | - | - | - | - | - | - | - | - | - | - | - | - | - |
| Nishida et al., 2023 | - | - | - | - | - | - | - | - | - | - | - | - | - | - | - | - | - | - |
|  | - | - | - | - | - | - | - | - | - | - | - | - | - | - | - | - | - | - |

*TG – Total gastrectomy, PG – Partial gastrectomy, DG – Distal gastrectomy, CG – Control group, IG – Intervention group*
